# 360° Virtual Reality: A SWOT Analysis in Comparison to Virtual Reality

**DOI:** 10.3389/fpsyg.2020.563474

**Published:** 2020-10-07

**Authors:** Aden Kittel, Paul Larkin, Ian Cunningham, Michael Spittle

**Affiliations:** ^1^Institute for Health and Sport, Victoria University, Footscray, VIC, Australia; ^2^Maribyrnong Sport Academy, Melbourne, VIC, Australia; ^3^Faculty of Health Sciences, Ontario Tech University, Oshawa, ON, Canada

**Keywords:** virtual reality, immersive 360° video, decision making, representative learning design, sport

## Introduction

With advancement in technology and more interactive nature of the available technology, the use of applied technologies such as Virtual Reality (VR) is increasing at exponential rates in both academic and applied settings (Düking et al., 2018; Faure et al., 2020). VR is defined as simulations of a real or imaginary environment, where a participant can both perceive and interact with the environment (Craig, 2013). This presents virtual simulations in different formats such as flat or curved large screen displays, Cave Automatic Virtual Environment (CAVE) (where participants are in a room surrounded by a screen) and head mounted displays (HMD). Examples of VR include a baseball batting simulator (Gray, 2017) and virtual handball goalkeeper (Vignais et al., 2015) with the virtual simulation opponents designed using complex motion capture systems. Given the rise of this technology, Düking et al. (2018) recently assessed VR for use in athletes through a SWOT analysis (i.e., strengths, weaknesses, opportunities and threats) identifying VR as appropriate for certain sporting areas, but more development of technology is needed to be more realistic.

**Figure 1:**
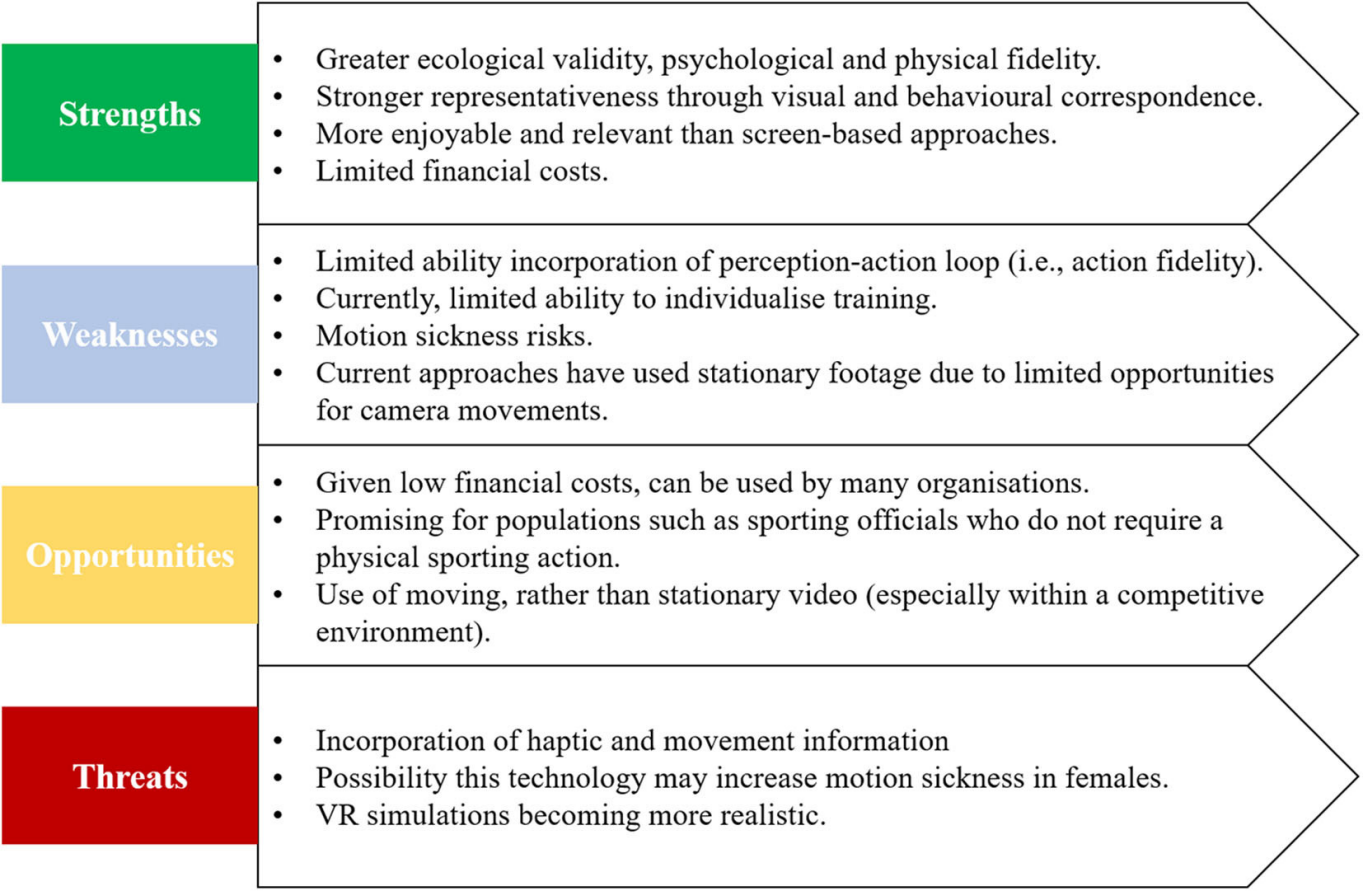
Summary of key findings from SWOT analysis.

A similar technology is 360°VR (also known as immersive video; Panchuk et al., 2018). Where VR involves virtual characters sourced through motion capture systems, 360°VR uses real-world footage filmed from a 360° camera. Both 360°VR and VR present the stimulus through a HMD, which allows the participant to scan, increasing the level of “presence” where the participants feel they are immersed in the environment (Slater, 2018; Bird, 2020). 360°VR has been labeled a suitable “middle ground” between VR and existing screen-based video occlusion technologies (Fadde and Zaichkowsky, 2018). This is because participants can scan in the 360° environment, but as they are watching real world video, cannot interact with the environment as in VR. Although VR and 360°VR are similar technologies, this paper highlights the distinct differences between the two, given their contrasting strengths and weaknesses. Figure 1 briefly illustrates the strengths, weaknesses, opportunities, and threats of 360°VR technology.

In recent years, 360°VR has been examined as a method to both assess (Kittel et al., 2019a) and train sport-specific decision-making skill (Panchuk et al., 2018; Pagé et al., 2019; Kittel et al., 2020). Efficacious training of decision-making skills is imperative, as research indicates that decision making skills distinguish between elite and novice performers (Mann et al., 2007; Kittel et al., 2019b).

## Strengths

As 360°VR uses real-world footage rather than virtual scenarios, decision-making is more realistic than VR. Research has demonstrated a higher level of perceived game-likeness in decision-making processes of 360°VR than more common screen-based approaches (e.g., match broadcast video) (Kittel et al., 2019a). This infers greater ecological validity of 360°VR as the perceptual information is more similar to the competitive environment (Araujo et al., 2007). Tasks with stronger representativeness and/or ecological validity elicit more significant expert-novice differences and lead to stronger transfer effects of training to gameplay (Farrow et al., 2018; Hadlow et al., 2018). Given the stronger game-likeness and ecological validity, participants view 360°VR to be a more enjoyable and relevant training tool than existing screen-based approaches (Kittel et al., 2020). This is an important consideration given high rates of burnout and dropout in competitive youth sport (Eime et al., 2019).

From a theoretical viewpoint, 360°VR is more representative of the competitive environment due to the higher levels of fidelity, which is defined as how much a simulation replicates reality (Alessi, 1988; Farrow, 2013). Firstly, psychological fidelity refers to how life-like the simulation is perceived by the participants (i.e., higher game-likeness outlined above), and physical fidelity is the extent the simulation looks like the real competitive environment (Stoffregen et al., 2003; Lorains et al., 2013). The first-person viewpoint provides a more accurate representation of in-game perceptual information and higher levels of fidelity than third-person (Craig, 2013). Physical fidelity is achieved through real-world footage, which overcomes a significant weakness of VR using virtual environments (Düking et al., 2018).

The higher psychological and physical fidelity outlined above contributes to stronger visual correspondence of perceptual information received in-game (Pinder et al., 2015). This links to the concept of ecological validity above where simulations should be as game-like as possible in their presentation. Where there is stronger psychological fidelity, participants are more likely to have similar gaze behaviors and attentional focus to certain cues (Gray, 2019). Behavioral correspondence is another important consideration for representative tasks (Pinder et al., 2011; Hadlow et al., 2018). This is achieved through the head movements, which unlike screen-based technology, automatically update when wearing a HMD to scan the 360° environment (Craig, 2013). As Craig (2013) outlines, head movements afforded through a HMD do not disrupt the optic flow similar to actual competition.

Theoretically, the stronger representativeness and ecological validity have led to some positive improvements following training studies (Panchuk et al., 2018; Pagé et al., 2019; Kittel et al., 2020). These studies, however, are preliminary methods and can be expanded on in future studies to further investigate the effectiveness of this technology.

The strengths of 360°VR technology overcome significant weaknesses of VR technologies such as creating realistic environments and the financial development costs. As discussed by Panchuk et al. (2018), most sporting organizations (e.g., lower-budget youth, amateur, sub-elite) do not have the financial capacity to hire external software developers to design virtual content.

## Weaknesses

When designing perceptual-cognitive tasks, it is important to consider perception-action coupling (Craig, 2013; Hadlow et al., 2018). For example, an athlete might need to intercept a ball (Brault et al., 2015) or tackle an opposition player (Brault et al., 2012). By incorporating perception-action coupling, this is more naturalistic and representative of the performance environment, allowing for more realistic training opportunities than pointing at a screen or verbalizing their response (Craig, 2013). This is an example of action fidelity, defined as the similarity between the participant's physical response/action between the off-field (experimental) and on-field (performance) settings (Pinder et al., 2011). Incorporating perception-action coupling into 360°VR is more difficult than VR, given participants are viewing pre-recorded video rather than interacting within a virtual space. As such, 360°VR has been labeled as “read-only” (Fadde and Zaichkowsky, 2018). This has led to most studies requiring a verbal response when making a decision (Kittel et al., 2019a, 2020; Pagé et al., 2019). Panchuk et al. (2018) mimicked a motor response by requiring basketballers to hold a basketball, and then imitate a passing or shooting action at the point of the decision.

In contrast to the ability of VR to freely manipulate scenarios to individualize training (Düking et al., 2018; Faure et al., 2020), 360°VR currently has limited capability to do so. In current training studies (Panchuk et al., 2018; Pagé et al., 2019; Kittel et al., 2020), there is a “one-size fits all” approach without the individualization of training.

Motion sickness may be an issue, causing one participant to drop out of a study (Panchuk et al., 2018). Although each of the 360°VR training/testing studies outlined have used stationary footage, expensive high stability systems are required if there is a moving camera (Litleskare and Calogiuri, 2019). Recent studies have required participants to sit down while watching 360°VR to avoid motion sickness (Pagé et al., 2019; Kittel et al., 2020). Although Panchuk et al. (2018) allowed participants to stand up, they were constrained to remaining in the same place. When participants are viewing 360°VR through a HMD, they can only view what is in their field of view on the HMD, not their immediate environment. As current technology does not allow participants to move within a 360°VR environment, researchers and practitioners should consider presenting scenarios that do not require dynamic movement from the participant. For example, 360°VR simulations may involve participants scanning for information around them, rather than moving to intercept a ball or opponent such as VR (Brault et al., 2012). The limited amount of movement in 360°VR to VR is a consideration that future technological developments may overcome.

When developing decision-making for basketball athletes, studies have used scripted plays (Panchuk et al., 2018; Pagé et al., 2019). Although this may be an effective and efficient way to capture the required videos, this may not be the most representative way to capture scenarios in other invasion sports, such as Australian football or Rugby, that involve significant physical contact and may be difficult to simulate. This can be overcome by capturing videos of competitive small-sided game activities (Kittel et al., 2019a), but no scripting leads to other limitations, such as limited clip scenarios and a large bank of videos.

Finally, the videos required for 360°VR are significantly larger in file storage size than screen-based video such as match footage. If plays are not scripted when recording, this requires a large bank of 360°VR videos with significant file sizes. Although software is constantly developing to accommodate large file sizes, researchers and practitioners must be aware of the storage limitations associated.

## Opportunities

A significant opportunity for 360°VR is the limited financial costs of this technology in comparison to VR (Düking et al., 2018). 360°VR technology is freely available from retail stores at accessible prices (Panchuk et al., 2018). Only elite organizations may have the financial capacity to afford VR systems, 360°VR is more available to a wider range of sporting organizations at sub-elite, amateur and youth levels.

As highlighted above, one of the limitations of 360°VR is that it is “read-only” (Fadde and Zaichkowsky, 2018), where this technology may limit the perception-action loop. This presents an opportunity for a considerable market such as sporting officials around the globe. Sporting officials do not complete a motor action such as a pass or intercept, but verbalize their decision. This has led to research interest in the area of sporting officials such as Australian football umpires, demonstrating promising findings of this technology (Kittel et al., 2019a, 2020). As Australian football umpires have high movement within a dynamic environment and 360°VR is currently captured with a stationary camera, this technology may be more beneficial for less dynamic officials such as tennis or cricket umpires. This may open the avenue to sporting officials' organizations across the globe to test and implement 360°VR technology for training and development.

Developing technology can greatly assist 360°VR to progress in coming years. Given current studies have used stationary footage in their methods (Panchuk et al., 2018; Kittel et al., 2019a; Pagé et al., 2019), a more representative method would be to implement moving footage as athletes and officials typically make decisions while moving. Further investigation of stabilizing techniques and the potential impact on motion sickness (Litleskare and Calogiuri, 2019) is a consideration for sport-related research.

Where suitable, methods may look to capturing first person 360°VR in competitive game scenarios. As this is competitive performance rather than mock drills or small-sided games, this would increase fidelity. This would allow for greater levels of presence, where participants are immersed within their natural competitive environment (Slater, 2018). Adopting this approach would optimize the visual correspondence and therefore representativeness of this technology (Hadlow et al., 2018). Use of 360°VR first-person game footage may allow other forms of training such as reflective learning, similar to existing protocols in education (Walshe and Driver, 2019).

Finally, 360°VR may include haptic and movement information such as vibrations and noise, similar to VR approaches (Düking et al., 2018). This would strengthen the representativeness of this technology.

## Threats

Immersive environments such as 360°VR and VR have the ability to cause motion sickness (Litleskare and Calogiuri, 2019). Research indicates VR induces motion sickness more in females (Munafo et al., 2017). Future studies may consider whether there is a similar effect in 360°VR. It should be noted females have effectively used this technology in previous training studies (Panchuk et al., 2018; Pagé et al., 2019), suggesting it may be appropriate to use. Future studies should investigate whether any gender-based differences exist for motion sickness in 360°VR. If motion sickness has a significant impact, researchers and practitioners must explore the financial trade-off of expensive video stabilizing technology (Litleskare and Calogiuri, 2019).

Fadde and Zaichkowsky (2018) outline that there are sometimes conflicting goals of the sport scientist and the coach/athlete. For example, sport scientists consider the validity of tools such as 360°VR, yet it is important to consider the financial cost, complexity and degree to which athletes and coaches accept new applied technologies. The battle to win acceptance by coaches and athletes is akin to other technologies, similar to VR (Düking et al., 2018).

Further refinement of VR approaches may lead to virtual simulations being more realistic, which is a current limitation of VR (Düking et al., 2018). With technological advancements potentially making VR more realistic, 360°VR may no longer be considered an effective option. Therefore, 360°VR should continue to progress to allow movement and include features to increase realism such as noise and haptic feedback.

## Summary

In summary, 360°VR appears to be a promising applied technology for assessing and developing decision-making skill in sport. Given decision-making skill has the ability to distinguish between performance levels of athletes (Mann et al., 2007) and officials alike (Kittel et al., 2019b), research must refine methods to develop decision-making skill. Significantly, 360°VR may be considered a more representative tool given the theoretical underpinning outlined in this paper (Hadlow et al., 2018). This SWOT analysis should outline for practitioners whether 360°VR may be a suitable applied technology for their athletes to use in developing their decision making skill. Practitioners and researchers should be aware of the limitations outlined, with the possibility that technological advancements may overcome some of the present limitations.

As outlined by Düking et al. (2018), SWOT analyses have their limitations and are subjective in nature. However, it is anticipated the findings of this paper will assist researchers and practitioners in determining the suitability and feasibility of 360°VR for their chosen sport. 360°VR may be an attractive applied technology for training decision-making skills at all sporting levels including elite, high performance youth and amateur, given the financial accessibility in comparison to more expensive VR technologies.

## Author Contributions

AK wrote the article. PL, IC, and MS all helped with the conceptual idea and editing.

## Conflict of Interest

The authors declare that the research was conducted in the absence of any commercial or financial relationships that could be construed as a potential conflict of interest.
